# High MAL2 expression predicts shorter survival in women with triple-negative breast cancer

**DOI:** 10.1007/s12094-024-03514-4

**Published:** 2024-05-20

**Authors:** Jędrzej Borowczak, Marek Zdrenka, Weronika Socha, Karol Gostomczyk, Krzysztof Szczerbowski, Mateusz Maniewski, Hanna Andrusewicz, Joanna Łysik-Miśkurka, Tomasz Nowikiewicz, Łukasz Szylberg, Magdalena Bodnar

**Affiliations:** 1 Department of Tumor Pathology and Pathomorphology, Oncology Centre, Prof. Franciszek Łukaszczyk Memorial Hospital, Bydgoszcz, Poland; 2 Department of Obstetrics, Gynaecology and Oncology, Collegium Medicum in Bydgoszcz, Nicolaus Copernicus University in Toruń, Bydgoszcz, Poland; 3 Doctoral School of Medical and Health Sciences, Nicolaus Copernicus University in Toruń, Bydgoszcz, Poland; 4 Clinical Department of Breast Cancer and Reconstructive Surgery, Oncology Center, Prof. Franciszek Łukaszczyk Memorial Hospital, Bydgoszcz, Poland; 5 Department of Surgical Oncology, Collegium Medicum in Bydgoszcz, Nicolaus Copernicus University in Toruń, Bydgoszcz, Poland; 6 Chair of Pathology, University Hospital No. 2 im. Dr. Jan Biziel in Bydgoszcz, Ujejskiego 75, 85-168 Bydgoszcz, Poland

**Keywords:** Breast cancer, Triple-negative, Prognosis, Survival, Biomarker, MAL2, PD-L1

## Abstract

**Introduction:**

Due to its lack of conventional surface receptors, triple-negative breast cancer (TNBC) is inherently resistant to most targeted therapies. MAL2 overexpression prompts endocytosis, conferring resistance to novel therapeutics. This study explores the role of MAL2 and PD-L1 in TNBC patients’ prognosis.

**Methods:**

We performed immunohistochemical analysis on 111 TNBC samples collected from 76 patients and evaluated the expression of MAL2 and PD-1. We expanded the study by including The Cancer Genome Atlas (TCGA) cohort.

**Results:**

MAL2 expression did not correlate with stage, grade, tumor size, lymph node invasion, metastasis, and PD-1 expression. Patients with high MAL2 had significantly lower 5-year survival rates (71.33% vs. 89.59%, *p* = 0.0224). In the tissue microarray cohort (TMA), node invasions, size, recurrence, and low MAL2 (HR 0.29 [CI 95% 0.087–0.95]; *p* < 0.05) predicted longer patients’ survival. In the TCGA cohort, patients with low MAL2 had significantly longer overall survival and disease-specific survival than patients with high MAL2. Older age and high MAL2 expression were the only independent predictors of shorter patient survival in the BRCA TCGA cohort.

**Conclusion:**

High MAL2 predicts unfavorable prognosis in triple-negative breast cancer, and its expression is independent of PD-1 levels and clinicopathological features of TNBC.

**Supplementary Information:**

The online version contains supplementary material available at 10.1007/s12094-024-03514-4.

## Introduction

Breast cancer is the most common female malignancy worldwide. Although overall survival has significantly increased over the past few years, it remains the second most prevalent cause of cancer-related deaths in women, accounting for 2.3 million new cases and 685,000 deaths in 2020 [1]. Triple-negative breast cancer (TNBC) accounts for approximately 15–20% of all breast cancer cases and lacks the expression of estrogen receptor (ER), progesterone receptor (PR), and human epidermal growth factor receptor 2 (HER2) [2]. This aggressive subtype of breast cancer affects predominantly young patients, especially those under 40 and African Americans, who rarely respond to standard targeted therapies and progress aggressively [2–4]. While early detection increases the chances of radical treatment and improves prognosis, current treatment modalities are limited to surgery, chemotherapy, radiation therapy, and only a few targeted therapies. Therefore, there exists a critical need to find new biomarkers and develop novel approaches to treat triple-negative breast cancer [5, 6].

Genetic and molecular factors play a key role in the development and progression of TNBC. Considering its inherent resistance to targeted therapies, developing novel prognostic markers became crucial to planning treatment, assessing disease progression, and stratifying patients’ risk [6]. Recently, T Cell Differentiation Protein 2 (MAL2) emerged as a potential driver of cancer immune escape and novel therapeutic target [7]. MAL2 was overexpressed in tumors and was associated with poor prognosis, and MAL2 knockdown improved major histocompatibility complex type 1 (MHC-1) recognition on tumor surfaces by CD8 + T cells, highlighting the role of MAL2 in immune regulation [8–11]. While the exact mechanism remains unknown, reducing MAL2 expression may be a novel therapeutic approach. More research is needed to fully understand the protein potential.

Targeting PD-1/PD-L1 has recently revolutionized patient care and started the era of immunotherapy in TNBC. PD-L1 is present on the surface of tumor cells and binds with Programmed Cell Death Protein 1 (PD-1) present on T cells, rendering them unable to induce antitumor response [12, 13]. Approximately 20% of TNBCs express Programmed Cell Death Ligand 1 (PD-L1), indicating their potential sensitivity to immune checkpoint inhibitors [14]. Blocking PD-1/PD-L1 interactions significantly improved the efficacy of cancer treatments in solid tumors, including breast cancer [15, 16]. While not all anti-PD-L1 regimens showed clinical benefits, blocking this interaction remains a promising treatment option [17]. Despite their distinct mechanisms of action, MAL2 and PD-1/PD-L1 expression suppress immune surveillance and facilitate cancer progression [9, 13]. Therefore, their concurrent targeting may hold the premise of improving therapeutic strategies to treat TNBC and prolong patient survival.

In this study, we explore the role of MAL2 in TNBC and investigate its interactions with PD-1 and PD-L1. Since both MAL2 and PD-L1 modulate tumor immune response, confirming their interactions may uncover the role of MAL2 as a novel prognostic marker and therapeutic target in TNBC.

## Materials and methods

### Sample acquisition

This study included 111 triple-negative breast cancer samples collected from 76 women in the Department of Pathomorphology between 2011 and 2015. Clinical data, including age, sex, overall survival, tumor differentiation (grade), stage T, lymph node invasion, metastasis, tumor size, and recurrence, were obtained and analyzed retrospectively (Table 1). The study followed the Declaration of Helsinki, and the protocol was approved by the Nicolaus Copernicus University Bioethics Committee (KB 746/2021). All 76 patients were treated by surgery, and the resection was radical in 75 cases. In 57 cases, it was breast-conserving surgery; in 19 cases, radical mastectomy was conducted. Three patients underwent neoadjuvant chemotherapy. Of 73 patients qualified for adjuvant therapy, 24 underwent chemotherapy, 47 chemotherapy and radiotherapy, while 2 were radiotherapy only. Three patients were disqualified from adjuvant therapy due to the high risk of adverse events relative to comorbidities. The high and low MAL2 expression groups (extracted from the tissue microarray (TMA), TCGA breast cancer, and TCGA TNBC cohorts) did not differ significantly in regard to administered treatment (*p* > 0.05).

**Table 1:** Basic patient characteristics

Clinical data	*n* (%)
Cases	76
Samples	111
Median age	59.5 years (range 32–85)
Age	
≤ 60	41 (54.67%)
> 60	35 (45.33%)
Sex	
Female	76 (100%)
Male	0 (0%)
HT/HRT	
No	67 (90.54%)
Yes	7 (7.45%)
Neoadjuvant chemotherapy	
No	73 (96.05%)
Yes	3 (3.95%)
Adjuvant therapy	
No	3 (3.95%)
Yes	73 (96.05%)
Nottingham grading score	
G1	1 (1.32%)
G2	31 (40.79%)
G3	41 (53.95%)
Stage	
T1	16 (21.05%)
T2	56 (73.68%)
T3	2 (2.63)
T4	2 (2.63%)
Lymph nodes invasion	
N0	54 (71.05%)
N1–N3	22 (28.85%)
Metastasis	
M0	76 (100%)
M1	0 (0%)
Tumor size	
≤ 25 mm	45 (59.21%)
> 25 mm	30 (40.79%)
Recurrence	
No recurrence	64 (84.21%)
Recurrence	12 (15.79%)
Time to recurrence (mean)	20.5 months (range 8–66)
Survival status	
Alive	57 (75%)
Dead	19 (25%)
Median follow-up time	72 months (range 2–72 months)

### Immunohistochemistry

A retrospective immunohistochemical analysis of MAL2 comprised 111 formalin-fixed, paraffin-embedded tissue blocks cut into 5 μm sections, attached to a glass slide, and incubated at 60 °C for 2 h. IHC staining was performed on the Ventana Benchmark Ultra platform according to NordiQC operating procedure. A primary MAL2 polyclonal antibody (ab75347, Abcam) was used for staining. The specimens were fixed in 10% buffered formalin for 24 h at room temperature.

### Image acquisition and analysis

The pathologists evaluating the immunohistochemical expression of PD-L1 worked independently and were blinded to clinical and pathological data. Protein expression was evaluated using a light microscope at 20 × original objective magnification. All of the collected tissue samples were processed following the standard diagnostic protocol. For this study, representative material from diagnostic biopsies and matching resection specimens was selected for additional immunohistochemical studies. The immunohistochemical studies of PD-L1 expression were performed using an anti-PD-L1 antibody (clone SP142, Ventana Medical Systems) on material from diagnostic biopsies, and the original protocol provided by Ventana was followed. Finally, the brown color due to the histochemical reaction product was considered as observed in the site of the presence of the searched antigen. According to the guide provided by Ventana, tumors with PD-L1 expression ≥ 1% IC were considered PD-L1-positive [18].

**Fig. 1:**
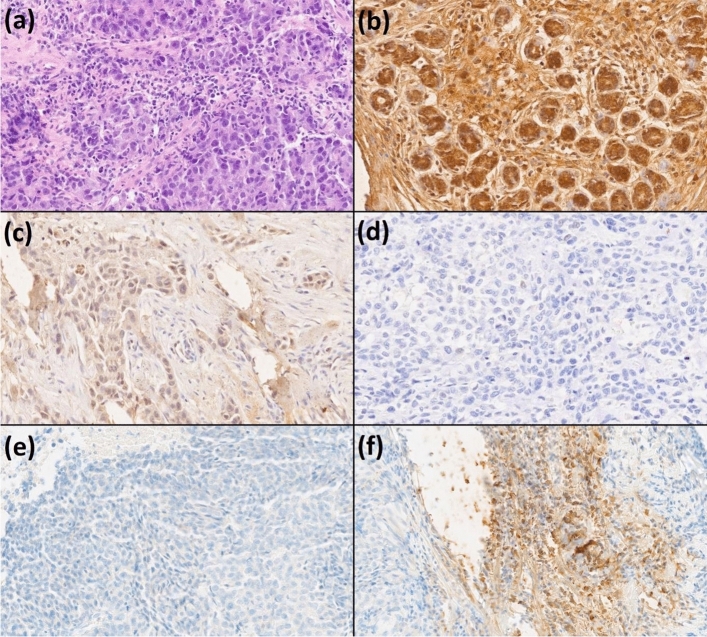
Cross-section TNBC staining patterns: **a** H&E, **b** high MAL2 expression, **c** low MAL2 expression, **d** negative MAL2 expression, **e** negative PD-L1 expression, and **f** positive PD-L1 expression

Matching resection specimens were stained to assess MAL2 expression. For each sample, the expression of MAL2 was obtained by calculating the H-score in three representative areas of the tumor. The H-score was assigned using the formula [1 × (% cells low positive) + 2 × (% cells positive) + 3 × (%cells high positive)], obtaining a value from 0 to 300. The final result was the mean value of the three assessments [19] (Fig. 1).

### In silico analysis

TCGA cohort clinical data were accessed through the cBioPortal [20]. USCS XENA was used to acquire normalized expression data of MAL2 and PD-1 [21]. FPKM gene expression was obtained through the Human Protein Atlas [22, 23]. Normalized gene expression was calculated as log2 (fpkm + 1). The cutoff was set at 126.57 FPKM for breast cancer and at 136.7 FPKM for TNBC.

### Statistical analysis

All statistical analyses were performed using Statistica version 13.3 (Statsoft) and Microsoft Excel 2019. *p* value < 0.05 was considered statistically significant. Variables were tested for normality using the Kolmogorov–Smirnov test. The intergroup comparisons used the Mann–Whitney *U* test or the ANOVA Kruskal–Wallis test. The correlations between MAL2 expression and the clinicopathological features of triple-negative bladder cancer were evaluated using Spearman’s rank correlation coefficient. Univariate and multivariate analyses of potential predictors for overall survival implemented the Cox proportional hazard regression. The optimal cutoffs were calculated using Cutoff Finder [24] Results were expressed as hazard ratio (HR) and 95% confidence interval (CI). The estimation of survival differences between groups was based on the log-rank test.

## Results

### MAL2 expression is independent of clinical features of TNBC

First, we calculated the expression of MAL2 depending on the clinical features of TNBC (Table 2, Table S1). MAL2 did not correlate with stage, grade, tumor size, and lymph node invasion. Its expression was independent of age, PD-L1 status, Ki-67 status, progression, and recurrence. Although the PD-L1 status and lymph node invasions show a trend toward higher MAL2 expression, those correlations were not statistically significant. There was no correlation between MAL2 expression and received neoadjuvant or adjuvant therapy (*p* > 0.05).

**Table 2:** MAL2 expression based on clinical features

Clinical data	Total (*N*)	Median MAL2 expression	Q1	Q3	*p* value
≤ 60 years	59	110 (range 275–84)	84	185	0.772
> 60 years	52	111 (range 0–265)	77.5	167.5	
HRT	97	112 (range 0–275)	90	180	0.22
No HRT	10	87 (range 0–260)	15	160	
IA	24	110 (range 0–265)	80	200	0.1
IIA	72	110 (range 0–265)	75	162	
IIB	10	182 (range 70–275)	114	230	
IIIB	5	105 (range 0–127)	105	105	
T1	20	110 (range 0–265)	92	150	0.446
T2	84	112 (range 0–275)	82	180	
T3	4	70 (range 32–112)	41	101	
T4	3	105 (range 105–127)	105	127	
N0	78	105 (range 0–265)	70	160	0.08
N1–N3	33	114 (range 0–275)	101	200	
NGS1	2	95 (range 10–180)	10	180	0.742
NGS2	48	110 (range 0–265)	77	170	
NGS3	56	112 (range 0–275)	88	187.5	
≤ 25 mm	62	119 (range 0–275)	86	185	0.268
> 25 mm	47	105 (range 0–260)	70	165	
No recurrence	91	110 (range 0–275)	80	180	0.9175
Recurrence	20	111 (range 0-230_	95	139.5	
PD-1 negative	61	105 (range 0–255)	70	160	0.072
PD-1 positive	49	125 (range 00–275)	95	190	
Low Ki-67	17	135 (range 5–275)	102	200	9.208
High Ki-67	35	110 (range 5–240)	80	170	

*NGS* Nottingham grade score; *T* stage; *N* lymph node involvement; *M* distant metastasis; *HRT* history of hormone replacement therapy

### MAL2 predicts shorter survival in TNBC

**Fig. 2:**
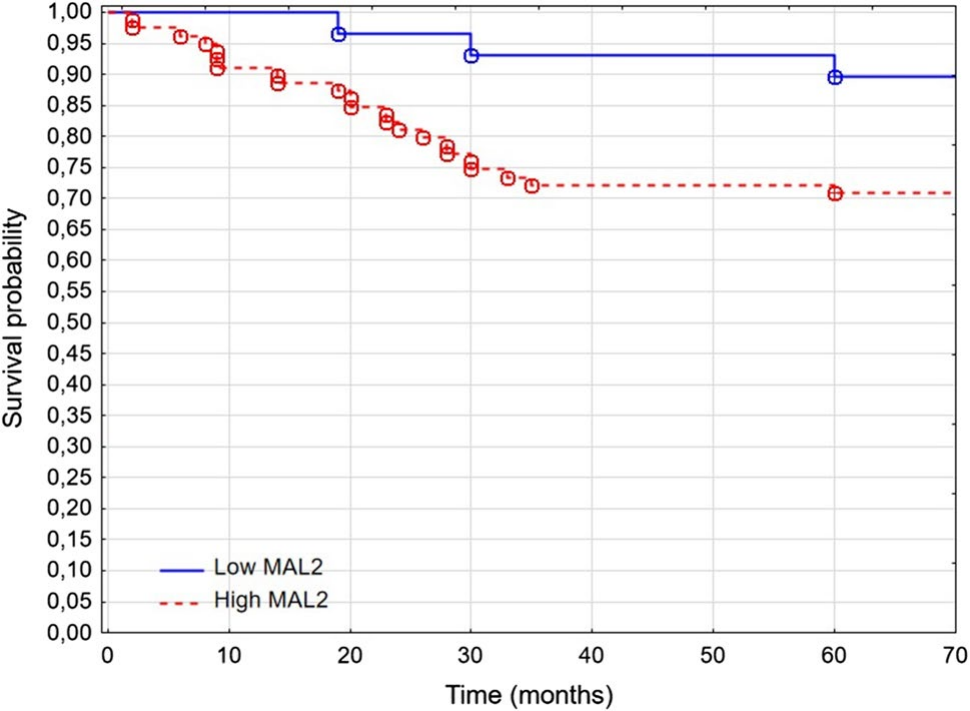
Patients’ survival depends on MAL2 status (89.59% vs. 71.33% 5 years after diagnosis in low-MAL2 and high-MAL2 groups, respectively; *p* = 0.0244)

To examine the predictive value of MAL in patients with triple-negative breast cancer, we dichotomized the samples into low and high MAL2 expression groups, with the cutoff point set at 88 H-score. H-score ≤ 88 indicated low MAL2 expression, and H-score > 88 indicated high MAL2 expression. Patients with high MAL2 had significantly lower 5-year survival rates (71.33% vs. 89.59%, respectively; *p* = 0.0224) than patients with low MAL2 (Fig. 2). The Kaplan–Meier analysis of overall survival by quartiles showed significant differences in OS between patients in the first and second quartiles of MAL2 expression (*p* = 0.021). MAL2 expression did not predict disease-free survival in patients with TNBC (86.75% vs. 81.59% 5 years after diagnosis; *p* = 0.41).

In univariate Cox regression analysis, lymph node invasions, size, recurrence, and low MAL2 expression (HR 0.29 [CI 95% 0.087–0.95]; *p* < 0.05) were associated with longer patients’ survival. In multivariate analysis, only disease recurrence and lymph node invasion retained statistical significance (Table 3). Given its significance in univariate analysis, we expand the analysis of MAL2 onto the TCGA cohort.

**Table 3:** Univariate and multivariate analysis of survival prognosis factors in TNBC

Variable	Univariate analysis			Multivariate analysis		
	HR	95% CI	*p* value	HR	95% CI	*p* value
Age (≤ 60 vs. > 60)	1.11	0.53–2.35	0.78	–	–	–
Stage (T1 vs. T2–T4)	0.295	0.07–1.24	0.097	–	–	–
Nodes (N0 vs. N1–N3)	**0.2**	**0.095–0.433**	**0.00004**	**0.358**	**0.158–0.815**	**0.014**
NGS (G1 vs. G2–G3)	0.787	0.36–1.71	0.55	–	–	–
Size (≤ 25 mm vs. > 25 mm)	**0.4**	**0.186–0.851**	**0.017**	**0.527**	**0.23–1.19**	**0.12**
Recurrence (no vs. yes)	**0.1**	**0.046–0.22**	**0.0000001**	**0.18**	**0.07–0.435**	**0.00014**
PD-1 (negative vs. positive)	1.485	0.685–3.219	0.316	–	–	–
Ki-67 (low vs. high)	1.128	0.385–3.3	0.826	–	–	–
MAL2 (low vs. high)	**0.29**	**0.087–0.95**	**0.041**	**0.417**	**0.124–1.406**	**0.158**

Sstatistically significant results are bolded

**Fig. 3:**
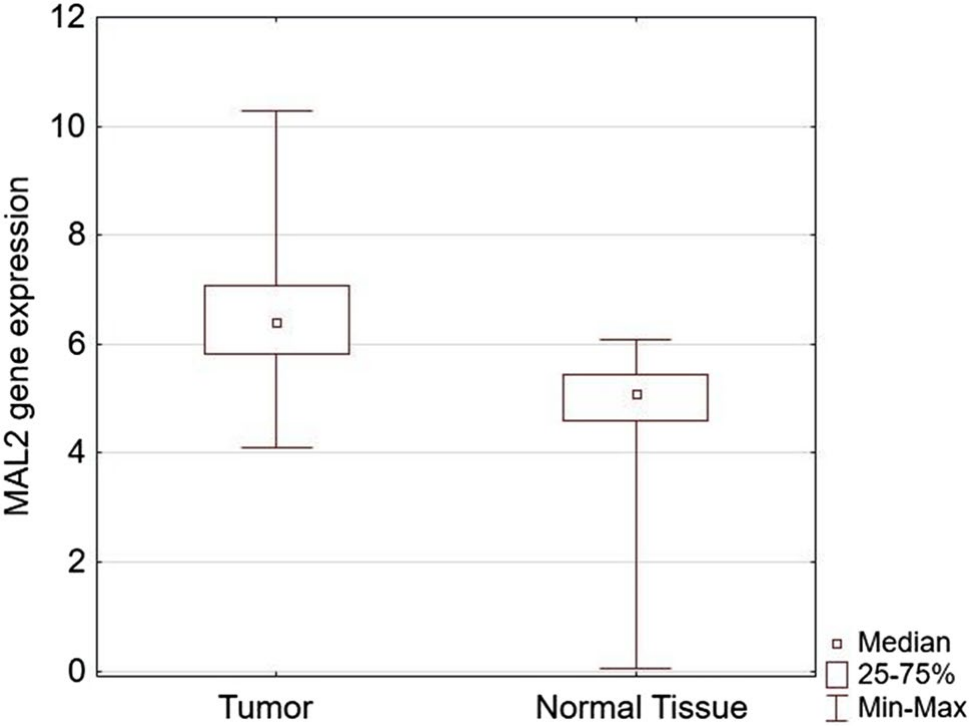
MAL2 and PD-1 are overexpressed in cancers compared to normal adjacent tissue (*p* = 0.0001 and *p* = 0.0007, respectively)

### In silico analysis

We assessed the expression of MAL2 and PDCD1 in the TCGA BRCA cohort using the XENA browser (Table 4) [22, 23, 25]. The normalized gene expression for MAL (5.09 vs. 6.4; *p* < 0.000001) and PDCD1 (0.29 vs. 0.37; *p* = 0.00049) was higher in tumors than in adjacent normal tissues. MAL2 and PDCD1 are overexpressed in breast cancer (Fig. 3).

**Table 4:** Basic characteristics of the TCGA cohorts

Clinical data	Breast cancer (%)	TNBC (%)
Cases	1052	169
Median age	58 (range 26–90)	57 (range 29–90)
Age		
≤ 60	586 (55.7%)	95 (56.21%)
> 60	466 (44.3%)	74 (43.79%)
Sex		
Female	1040 (98.86%)	165 (97.63%)
Male	12 (1.14%)	4 (2.37%)
Race		
White	743 (70.63%)	154 (91.12%)
Black or African-American	179 (17.02%)	10 (5.91%)
Asian	58 (5.51%)	5 (3.97%)
Neoadjuvant therapy		
Yes	6 (0.6%)	2 (1.18%)
No	1046 (99.4%)	167 (98.82%)
Adjuvant radiotherapy		
Yes	538 (51.14%)	72 (42.6%)
No	429 (40.78%)	84 (49.6%)
Unknown	85 (8.08%)	13 (7.69%)
Stage		
T1	117 (11.12%)	32 (18.93%)
T2	600 (57.03%)	91 (53.85%)
T3	239 (22.72%)	41 (24.26%)
T4	19 (1.8%)	3 (1.18%)
Lymph nodes invasion		
N0	497 (47.24%)	80 (47.34%)
N1–N3	536 (50.95%)	89 (52.66%)
Metastasis		
M0	872 (82.89%)	143 (84.62%)
M1	21 (2%)	4 (2.37%)
Progression		
No progression	907 (86.22%)	144 (85.21%)
Progression	145 (13.78%)	25 (14.79%)
Progression-free survival (median)	25.78 months (range 003–281)	28 months (range 0–143)
Follow-up time (median)	28.08 months (range 0.03–283)	30 months (range 0–130)
Survival status		
Alive	901 (85.65%)	144 (85.21%)
Dead	151 (14.35%)	25 (14.79%)

**Fig. 4:**
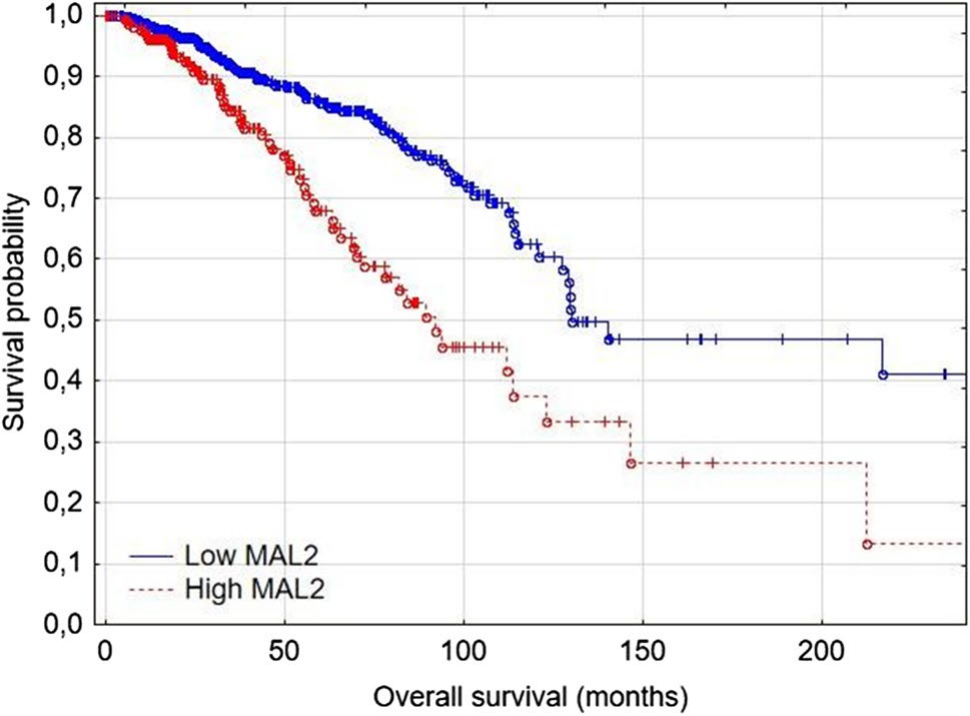
Overall survival in the TCGA cohort based on MAL2 expression (*p* = 0.0001)

We used the Ensembl gene ID from TCGA to map the TCGA RNA-seq data. The samples were dichotomized into low and high MAL2 expression groups based on the FPKMs value (number of fragments per kilobase of exon per Million reads).

Breast cancer patients with low MAL2 had significantly longer overall survival (*p* = 0.0001) and disease-specific survival (*p* = 0.0026) than patients with high MAL2 expression (Fig. 4). In multivariate COX regression analysis, older age and high MAL2 expression were the only independent predictors of shorter patient survival in the BRCA TCGA cohort (Supplementary materials).

**Fig. 5:**
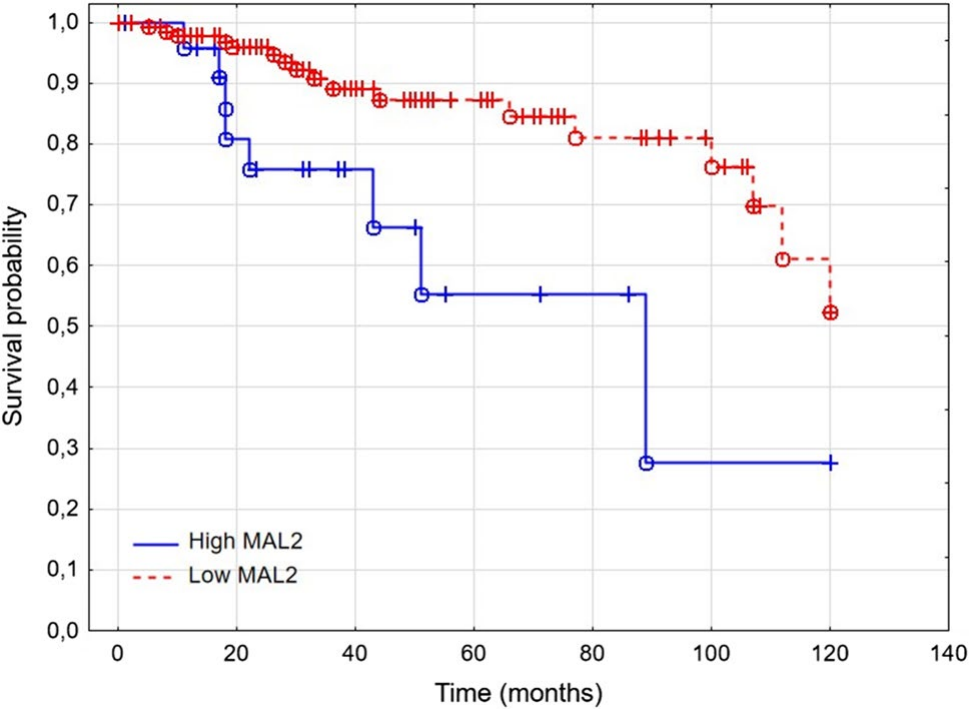
Overall survival in the TCGA TNBC cohort based on MAL2 expression (*p* = 0.008)

Next, we analyzed the role of MAL2 in a subset of TNBC patients extracted from the TCGA database. In the TNBC TGCA cohort, patients with high MAL2 expression had significantly higher 5-year survival than patients with low MAL2 expression (87.25% vs. 55.45%, respectively) (Fig. 5). Furthermore, high MAL2 expression, older age, higher stage, and disease progression were independent prognostic factors of shorter TNBC patients survival (*p* < 0.05) (Table 5). The results obtained from the TCGA cohort are consistent with the findings from our tissue microarray (TMA) cohort.

**Table 5:** Univariate and multivariate analysis of the TCGA triple-negative breast cancer cohort

Variable	Univariate analysis			Multivariate analysis		
	HR	95% CI	*p* value	HR	95% CI	*p* value
Age (≤ 60 vs. > 60)	2.63	1.16–5.99	**0.02**	**3.41**	**1.34–8.66**	**0.01**
Sex (male vs. female)	0.34	0.04–2.58	0.3	–	–	–
Stage (T1–T2 vs. T3–T4)	**3.4**	**1.52–7.6**	**0.003**	**4.5**	**1.28–15.83**	**0.01**
Nodes (N0 vs. N1–N3)	**2.61**	**1.09–6.27**	**0.03**	1.27	0.36–4.48	0.71
Metastasis (M0 vs. M1)	**6.83**	**2.28–20.49**	**0.0006**	0.77	0.2–2.94	0.7
Progression (no vs. yes)	**9.48**	**4.05–22.23**	**0.00001**	**17.54**	**5.51–55.84**	**0.0001**
MAL2 (low vs. high)	**3.01**	**1.33–7.22**	**0.008**	**8.72**	**2.64–28.87**	**0.0003**

Statistically significant results are bolded

## Discussion

In our study, MAL2 overexpression was associated with shorter survival in triple-negative breast cancer. Although the results from the TCGA database analysis align with the results from our TMA cohort, MAL2 expression seems independent of PD-L1 status, tumor grade, stage, size, lymph node invasion, and metastasis. The lack of association with clinicopathological features of TNBC indicates that MAL2 independently contributes to cancer progression. However, the unclear relationship between MAL2, PD-1, and cancer immune escape needs to be addressed.

T Cell Differentiation Protein 2 (MAL2) is a 176 amino acid residue protein encoded by a gene located on chromosome 8 (8q24.12) [10, 11, 26]. It belongs to the MAL proteolipid family and encodes a transmembrane protein that participates in transcytosis [8, 27]. While the nature of the interactions between MAL2 signaling and breast cancer remains largely unexplored, recent studies have shed new light on its role in cancer immune escape [9]. Cancer cells interact with each other and modulate the adjacent stroma cells to form an immunosuppressive microenvironment (TME) where they dwell and proliferate [28]. In their niche, cells lose epithelial phenotype and acquire mesenchymal-like phenotype. This phenomenon, called epithelial–mesenchymal transition (EMT), is the key event that leads to cancer immune escape [29].

MAL2 is overexpressed in breast cancer, and its high expression correlates with poor prognosis [27]. The Bhandari et al. study showed that MAL2 knockdown decreased breast cancer cell proliferation, migration, and invasion. It was associated with increased E-cadherin and Vimentin levels, indicating that MAL2 drives disease progression by EMT. Those results seem to be confirmed by Yuan et al. study, in which MAL2 deletion in ovarian cancer cells reduced their proliferation, migration, invasion, and EMT [11].

The expression of PD-L1 on tumor cells increases along disease progression due to cancer immune escape, epithelial–mesenchymal transition, or the upregulation of the JAK-STAT, MAPK, and PI3K-AKT signaling pathways [30–32]. Contrary to MAL2, TNBC expressing PD-L1 seems to be associated with better outcomes and is more likely to respond to neoadjuvant chemotherapy [33]. While direct interactions between PD-1/PD-L1 and MAL2 are currently unknown, they seem to regulate T cell reactivity and anticancer immune response [9, 34]. By interacting with MHC-I and RAB, MAL2 caused the degradation of MHC-I, promoted tumor antigen endocytosis, and suppressed their presentation. MAL2 depletion in a mice xenograft suppressed breast tumor growth and enhanced tumor-infiltrating CD8 + T cells cytotoxicity, presumably by facilitating tumor antigen presentation [9].

While direct interaction between MAL2 and PD-1/PD-L1 remains unknown, their overexpression appears to reflect different mechanisms of therapeutic resistance. PD-1/PD-L1 affects the activation of T cells, while MAL2 renders them ineffective in recognizing malignant cells [13, 27]. Considering that we found no direct relationship between PD-1 and MAL2 expression, we hypothesize that both mechanisms occur separately and MAL2 overexpression may limit the efficacy of anti-PD-1/anti-PD-L1 therapy.

## Conclusion

MAL2 is overexpressed in TNBC, and high MAL2 expression predicts shorter overall survival in triple-negative breast cancer in both the TMA and TCGA cohorts. MAL2 levels are independent of patients’ age, PD-1 status, Ki-67 status, hormonal therapy, progression, and recurrence status. We found no direct correlation between the expression of PD-L1 and MAL2, but both proteins partake in tumor immunoregulation and may be intertwined in mediating tumor immune escape. While targeting MAL2 appears promising, further trials are needed to examine its clinical applicability.

## Supplementary Information

Below is the link to the electronic supplementary material.

Supplementary file1 (DOCX 15 kb)

## Data Availability

The data presented in this study are available on request from the corresponding author. Due to ethical restrictions, they are not publicly available.
