# Supplementary material for: High MAL2 expression predicts shorter survival in women with triple-negative breast cancer
Source: Clin Transl Oncol. 2024 May 20;26(10):2549–58. doi: 10.1007/s12094-024-03514-4 (PMC11410892; doi:10.1007/s12094-024-03514-4)
Supplement: Supplementary file 1 — Supplementary file1 (DOCX 15 kb) [file 12094_2024_3514_MOESM1_ESM.docx]

Supplementary Materials

Table S1. Univariate and multivariate analysis of the TCGA breast cancer cohort

| Variable | Univariate analysis | | | Multivariate analysis | | |
| --- | --- | --- | --- | --- | --- | --- |
|  | HR | 95% CI | P-value | HR | 95% CI | P-value |
| Age (≤ 60 vs. >60) | 0.509 | 0.369-0.703 | 0.00004 | 0.41 | 0.258-0.66 | 0.00023 |
| Stage (T1 vs. T2-T4) | 0.455 | 0.27-0.766 | 0.003 | 0.578 | 0.255-1.31 | 0.19 |
| Nodes (N0 vs. N1-N3) | 0.451 | 0.315-0.644 | 0.00001 | 0.781 | 0.46-0.132 | 0.359 |
| Metastasis (M0 vs. M1) | 0.2 | 0.12-0.336 | 0.000001 | 1.6 | 0.35-7.28 | 0.546 |
| Recurrence (No vs. Yes) | 0.15 | 0.098-0.232 | 0.000001 | 0.15 | 0.012-1.12 | 0.06 |
| Progression (No vs. Yes) | 0.17 | 0.23-0.23 | 0.000001 | 0.941 | 0.126-7.03 | 0.952 |
| MAL2 (Low vs. High) | 0.462 | 0.332-0.644 | 0.000005 | 0.5 | 0.308-0.809 | 0.0048 |

Supplementary table 1. The distribution of MAL2 expression based on clinical TNBC stages

| Clinical stage | Total (N) | Median MAL2 expression | Q1 | Q3 | P-value |
| --- | --- | --- | --- | --- | --- |
| All cases | 111 | 110 (range 0-275) | 84 | 175 | - |
| IA | 24 | 110 (range 0-265) | 80 | 200 | p=0.1 |
| IIA | 72 | 110  (range 0-265) | 75 | 162 |  |
| IIB | 10 | 182 (range 70-275) | 114 | 230 |  |
| IIIB | 5 | 105 (range 0-127) | 105 | 105 |  |
